# Age at diagnosis and diagnostic delay across attention-deficit hyperactivity and autism spectrums

**DOI:** 10.1177/00048674231206997

**Published:** 2023-10-27

**Authors:** Rachael Knott, Olivia J Mellahn, Jeggan Tiego, Kathryn Kallady, Louise E Brown, David Coghill, Katrina Williams, Mark A Bellgrove, Beth P Johnson

**Affiliations:** 1 Turner Institute for Brain and Mental Health, School of Psychological Sciences, Monash University, Clayton, VIC, Australia; 2 School of Nursing, Midwifery and Paramedicine, Curtin University, Bentley, WA, Australia; 3 Department of Paediatrics, Faculty of Medicine, Dentistry and Health Sciences, The University of Melbourne, Parkville, VIC, Australia; 4 Department of Mental Health, The Royal Children’s Hospital, Parkville, VIC, Australia; 5 Neurodevelopment and Disability Research, Murdoch Children’s Research Institute, The Royal Children’s Hospital, Parkville, VIC, Australia; 6 Department of Developmental Paediatrics, Monash Children’s Hospital, Clayton, VIC, Australia; 7 Department of Paediatrics, Monash University, Monash Children’s Hospital, Clayton, VIC, Australia

**Keywords:** ADHD, autism, diagnosis, diagnostic delay, comorbidity

## Abstract

**Background:**

Despite the known benefits of accurate and timely diagnosis for children with attention-deficit hyperactivity disorder and autism spectrum disorders (autism), for some children this goal is not always achieved. Existing research has explored diagnostic delay for autism and attention-deficit hyperactivity disorder only, and when attention-deficit hyperactivity disorder and autism co-occur, autism has been the focus. No study has directly compared age at diagnosis and diagnostic delay for males and females across attention-deficit hyperactivity disorder, autism and specifically, attention-deficit hyperactivity disorder + autism.

**Methods:**

Australian caregivers (*N* = 677) of children with attention-deficit hyperactivity disorder, autism or attention-deficit hyperactivity disorder + autism were recruited via social media (*n* = 594) and the Monash Autism and ADHD Genetics and Neurodevelopment Project (*n* = 83). Caregivers reported on their child’s diagnostic process. Diagnostic delay was the mean difference between general initial developmental concerns and the child’s attention-deficit hyperactivity disorder and autism diagnosis.

**Results:**

Children with autism were significantly younger at autism diagnosis than the attention-deficit hyperactivity disorder + autism group (η_p_^2^ = 0.06), whereas children with attention-deficit hyperactivity disorder were significantly older at attention-deficit hyperactivity disorder diagnosis than the attention-deficit hyperactivity disorder + autism group (η_p_^2^ = 0.01). Delay to attention-deficit hyperactivity disorder and autism diagnosis was significantly longer in the attention-deficit hyperactivity disorder + autism group compared to attention-deficit hyperactivity disorder (η_p_^2^ = 0.02) and autism (η^2^ = 0.04) only. Delay to autism diagnosis for females with autism (η^2^ = 0.06) and attention-deficit hyperactivity disorder + autism (η^2^ = 0.04) was longer compared to males.

**Conclusions:**

Having attention-deficit hyperactivity disorder + autism and being female were associated with longer delays to diagnosis. The reasons for these delays and possible adverse effects on outcomes require further study.

Attention-deficit/hyperactivity disorder (ADHD) and autism spectrum disorder (autism) are prevalent neurodevelopmental disorders affecting 5.9% and 2–4% of children, respectively (Faraone et al., 2021; Li et al., 2022), and are recognised as commonly co-occurring (ADHD + autism; Grzadzinski et al., 2016; Zablotsky et al., 2020). The average age for ADHD diagnosis is between five and nine years (Caci et al., 2020; Efron et al., 2013; Parr et al., 2003; Sainsbury et al., 2023) and between three and six years for autism (Bent et al., 2015; Brett et al., 2016; Martinez et al., 2018; Sainsbury et al., 2023). The breadth of symptoms covered under the diagnostic umbrella of ADHD and autism results in significant phenotypic heterogeneity in symptom frequency and clinical severity (Levy et al., 2009; Musser et al., 2014). With no current validated biological markers for either disorder, diagnosis is based on developmental history and observation of behaviours relative to expectations for age and abilities (Charman and Gotham, 2013; Faraone et al., 2021).

Timely diagnosis and access to intervention are associated with better social, mental health, educational, and functional outcomes in ADHD and autism (Boland et al., 2020; Bradshaw et al., 2015; Fuller and Kaiser, 2020). However, families face numerous barriers when seeking a diagnosis and support, which include a lack of education and training for general practitioners, poor community knowledge or negative misconceptions associated with ADHD or autism diagnoses (Elder et al., 2016; French et al., 2018; Hamed et al., 2015), leading to delays in receiving a diagnosis. This time taken to receive a diagnosis is often referred to as *diagnostic delay*, although the definition can differ across studies and country of origin, leading to varying estimates. When diagnostic delay is defined as the time from first contact with a healthcare professional to diagnosis, previous reports estimate delays between one (World Federation for Mental Health (WFMH), 2004) and almost three years (Purper-Ouakil et al., 2007) for ADHD, and between two (Siklos and Kerns, 2007; Zuckerman et al., 2017) and 3.5 years (Crane et al., 2016) for autism, depending on the country. However, developmental concerns can be present at least a year before the first contact with a healthcare professional in America (Stevens et al., 2016) and 3.5 years before a diagnostic assessment in Australia (Boulton et al., 2023). When diagnostic delay includes this period of first concerns, estimates are up to four years for ADHD (Caci et al., 2020) and between three and five years for autism (Narcisa et al., 2013; Sivberg, 2003).

Atypical presentation, the presence of co-occurring ADHD and autism (Brett et al., 2016; Purper-Ouakil et al., 2007) and biological sex may play a role in longer delays to diagnosis (Efron et al., 2014; Grevet et al., 2006; Russell et al., 2011; Siklos and Kerns, 2007). When children have ADHD + autism, diagnostic delay for autism is 3.25 years, compared to 1.97 years for children with autism only (Stevens et al., 2016). Some suggest ADHD symptoms may diagnostically overshadow autism symptoms, e.g., hyperactivity is the most prominent behaviour (Kentrou et al., 2019; Miodovnik et al., 2015; Wallisch et al., 2020). Furthermore, females with ADHD tend to be older when they receive their diagnosis (Grevet et al., 2006), and females with autism tend to be diagnosed 14 months later than males (Kavanaugh et al., 2023). The difficulties identifying milder or atypical presentations appear to be heightened for females (Lockwood Estrin et al., 2020; Wright et al., 2015).

In Australia, Efron et al. (2013) and Bent et al. (2015) reported on age at diagnosis for ADHD (nine years old) and autism (four years old), but these studies lacked a discussion of how co-occurring ADHD and autism may impact these estimates. Boulton et al.’s (2023) work provides critical first steps in understanding diagnostic delay in a large Australian neurodevelopmental sample drawn from a public service. The average delay from time at first concerns to diagnostic assessment was 3.5 years, with diagnostic assessments occurring at 6.5 years on average. However, their results do not capture children who may have received their diagnosis through private fee-paying services. As three-quarters of children who see a paediatrician for their ADHD diagnosis are seen privately (Efron et al., 2013), the next step is to examine diagnostic delay in Australian children accessing all service types.

Although diagnostic delay has been examined in children with ADHD + autism in the context of an autism diagnosis (Stevens et al., 2016), no study has directly compared age at diagnosis and diagnostic delay for males and females across ADHD, autism and specifically, ADHD + autism, across all service types. This study aimed to address the paucity of research examining age at diagnosis and diagnostic delay in males and females with ADHD, autism and ADHD + autism by (1) examining differences in age at diagnosis for ADHD, autism and ADHD + autism groups; (2) exploring differences in the average length of diagnostic delay for children with ADHD, autism and ADHD + autism; and (3) investigating differences in diagnostic delay for males and females with ADHD, autism and ADHD + autism.

## Methods

### Participants

Participants were caregivers of 677 children aged between 1 and 16 years of age (M = 9.13 years, SD = 3.14) with a caregiver-reported diagnosis of ADHD (*n* = 310), autism (*n* = 154) or ADHD + autism (*n* = 213). Caregivers were 18 years or older and reported their child’s age at ADHD and autism diagnosis, child’s age at first general developmental concerns, and who gave the diagnosis(es) (e.g. psychologist, paediatrician). Children were 18 years or younger and received their diagnosis within the last six years to minimise retrospective bias. Caregivers were primarily recruited via social media Australia-wide (*n* = 594), with additional families coming from the Monash Autism and ADHD Genetics and Neurodevelopment (MAGNET) Project in Victoria, Australia (*n* = 83; Knott et al., 2021). See Supplementary Table S1 for recruitment numbers by Australian state. Recruitment was completed between 2019 and 2022.

Of the 796 participants who commenced consent and basic demographic information, 677 (85%) went on to complete questions on diagnostic process. Data were missing completely at random for the overall sample of 796 (Little’s MCAR test [Little, 1998], χ^2^ (20) = 19.67, *p* = 0.479) and study sample of 677 (Little’s MCAR test [Little, 1998], χ^2^ (68) = 29.10, *p* > 0.999).

A subset of the sample (*N* = 553) completed ADHD and autism symptom rating scales, the Conners’ Parent Rating Scale – Revised Long Form (CPRS-RL) and the Social Responsiveness Scale – Second Edition (SRS-2), as part of the survey. The ADHD + autism and autism groups showed significantly higher levels of autism symptoms, and ADHD and ADHD + autism groups showed higher levels of ADHD symptoms. Although symptom ratings reflect the child’s current ADHD and autism symptoms, these differences between diagnostic groups provide support for caregiver-reported diagnosis (see Appendix S2 for SRS-2 and CPRS-RL scores and Table S2 for significance testing).

### Ethical approval

The study was approved by the Monash University Human Research Ethics Committee (19721, 7504). Participants provided written informed consent.

### Measures

#### Diagnostic process

Caregivers completed questions regarding their family’s demographics, the child’s diagnostic process (age at first concerns, age at diagnosis), two symptom checklists for ADHD and autism symptoms indicating which behaviours were present when developmental concerns arose (yes/no), medication use, intervention engagement, caregiver satisfaction and stress throughout the diagnostic process, and current ADHD and autism symptom severity measures (see Appendix S3 for checklists). Questions pertaining to caregiver satisfaction and stress were adapted from Crane et al.’s (2016) study. The current study’s survey can be viewed here: https://figshare.com/s/60d8d9f739fd459271c9. Data on medication use and symptom severity are reported elsewhere (Mellahn et al., 2022).

#### Socio-economic status

Socio-economic status (SES) was estimated using the postcode provided by caregivers. The index of relative socio-economic advantage and disadvantage (IRSAD) from 2016 census data was used to group families into low (lowest 25%), middle (middle 50%) and high (highest 25%) SES categories (Australian Bureau of Statistics, 2018).

### Procedure

Questionnaires were administered online using Research Electronic Data Capture (REDCap; Harris et al., 2009). The caregivers completed one 60- to 90-minute survey for their child.

Diagnostic delay was the mean group difference in months between the age at initial general developmental concerns and age at diagnosis (either ADHD diagnosis or autism diagnosis). This approach was used for all children, resulting in two diagnostic delay values for the ADHD + autism group, one for ADHD and one for autism. Caregivers endorsed relevant co-occurring conditions for their child from a list and the number of these conditions were summed to create the ‘number of co-occurring conditions’ variable.

### Statistical analysis

Two one-way between-groups analysis of covariance (ANCOVAs) were performed to examine differences in age at ADHD diagnosis and age at autism diagnosis, with child sex, SES and number of co-occurring conditions entered as covariates. Two one-way between-groups ANCOVAs examine differences in delay to autism diagnosis and delay to ADHD diagnosis, with SES, child sex and number of co-occurring conditions as covariates. However, as homogeneity of variance could not be assumed for delay to autism diagnosis, *F* (1, 361) = 5.02, *p* = 0.026, a one-way between-groups analysis of variance (ANOVA) was performed using Welch’s *F* statistic for main effects (Field, 2013). Four one-way between-groups ANCOVAs were used to examine differences in delay to ADHD diagnosis and delay to autism diagnosis between males and females, within each diagnostic group, with SES and number of co-occurring conditions entered as covariates. However, as homogeneity of variance could not be assumed for delay to autism diagnosis within the autism, *F* (1, 151) = 8.36, *p* = 0.004, and ADHD + autism groups, *F* (1, 208) = 5.27, *p* = 0.023, and for delay to ADHD diagnosis in the ADHD + autism group, *F* (1, 306) = 8.92, *p* = 0.003, three one-way between-groups ANOVAs using Welch’s *F* statistic were performed (Field, 2013). Correlations between the covariates (SES, number of additional co-occurring conditions), age at ADHD and autism diagnosis, and delay to ADHD and autism diagnosis are presented in Supplementary Table S4.

As not all children with ADHD + autism receive their diagnoses at the same time, or in the same order, the ADHD + autism group was broken into three sub-groups for exploratory analysis: 1) children who received their ADHD diagnosis before autism (ADHD + autism – ADHD first); 2) children who received their autism diagnosis before ADHD (ADHD + autism – autism first); and 3) children who received their ADHD and autism diagnoses at the same time (ADHD + autism – same time). Two ANCOVAs were performed to examine differences in delay to ADHD and autism diagnoses between the three ADHD + autism subgroups. The findings of these exploratory analyses are presented in Appendix S8.

The Benjamini–Hochberg (1995) procedure was used to correct for multiple comparisons and control the false discovery rate. There were no interactions between covariates and dependent variables in completed ANCOVAs. Pairwise exclusion for missing data was used during analyses. Analyses were carried out using SPSS 27.

## Results

The 677 caregivers were predominantly biological mothers from Victoria and NSW, with ages ranging from 24 to 69 years (M = 39.47 years, SD = 6.78; see Table 1 for sample demographics). Most children received their diagnosis in a private setting, such as a private paediatrician (ADHD diagnosis = 83.70%, autism diagnosis = 78.60%), compared to a public setting, such as a public hospital clinic (ADHD diagnosis = 16.25%, autism diagnosis = 20.16%; see Supplementary Table S1 for a breakdown of diagnostic services). See Table 2 for mean age at first concerns, age at ADHD and autism diagnoses and diagnostic delay. An informal examination of the rates caregivers’ endorsed ADHD and autism symptoms at first concerns are presented in Appendix S3.

**Table 1.** Sample demographics for the families of children with ADHD, autism and ADHD + autism.

	ADHD *N* = 310	Autism *N* = 154	ADHD + autism *N* = 213	Total sample *N* = 677
Caregiver type				
Biological mother	278 (89.70%)	121 (78.60%)	172 (80.80%)	571 (84.30%)
Biological father	27 (8.70%)	31 (20.10%)	35 (16.40%)	93 (13.70%)
Adopted mother	3 (1.00%)	1 (0.60%)	1 (0.50%)	5 (0.70%)
Grandparent	0 (0.00%)	0 (0.00%)	1 (0.50%)	1 (0.10%)
Caregiver	1 (0.30%)	1 (0.60%)	3 (1.40%)	5 (0.70%)
Other	1 (0.30%)	0 (0.00%)	1 (0.50%)	2 (0.30%)
Caregiver age in years M(SD)	40.10 (6.86)	38.53 (6.47)	39.25 (6.82)	39.47 (6.78)
SES				
Low	35 (11.30%)	27 (17.50%)	39 (18.30%)	101 (14.90%)
Middle	154 (49.70%)	77 (50.00%)	109 (51.20%)	340 (50.20%)
High	121 (39.00%)	50 (32.50%)	64 (30.00%)	235 (34.70%)
Additional child diagnoses				
ID	4 (1.30%)	6 (3.90%)	14 (6.60%)	24 (3.5%)
Epilepsy	5 (1.60%)	0 (0.00%)	2 (0.90%)	7 (1.00%)
Learning disorder	48 (15.50%)	5 (3.20%)	19 (8.90%)	72 (10.60%)
ODD	55 (17.70%)	4 (2.60%)	39 (18.30%)	98 (14.50%)
Anxiety	81 (26.10%)	34 (22.10%)	0 (0.00%)	115 (17.00%)
Depression	11 (3.50%)	2 (1.30%)	16 (7.50%)	29 (4.30%)
S&L disorder	13 (4.20%)	6 (3.90%)	7 (3.30%)	26 (3.80%)
Genetic	2 (0.60%)	3 (1.90%)	7 (3.30%)	12 (1.80%)

ADHD: attention-deficit/hyperactivity disorder; Autism = autism spectrum disorder. ADHD + autism = co-occurring attention-deficit/hyperactivity disorder and autism spectrum disorder; Caregiver age = average in years (standard deviation); M: mean; SD: standard deviation; SES: socio-economic status; ID: intellectual disability; ODD: oppositional defiant disorder; SL: speech and language disorder.

Percentages are within group/ column percentages, e.g., 89.70% of caregivers in the ADHD group were biological mothers.

**Table 2.** Mean age in years at first developmental concerns, age at ADHD and autism diagnosis and average delay to ADHD and autism diagnosis.

	ADHD only *N* = 310	Autism Only *N* = 154	ADHD +autism *N* = 213
Age at first concern (M, SD)			
Males	3.95 (2.29)	2.22 (1.56)	2.69 (1.76)
Females	4.23 (2.29)	2.78 (2.57)	2.82 (1.91)
Total	4.02 (2.29)	2.39 (1.95)	2.72 (1.79)
Age at ADHD diagnosis (M, SD)			
Males	7.35 (2.33)		6.69 (2.24)
Females	7.97 (2.85)		7.57 (2.54)
Total	7.52 (2.49)		6.92 (2.35)
Age at autism diagnosis (M, SD)			
Males		4.96 (2.45)	6.61 (2.87)
Females		6.78 (3.46)	8.03 (3.08)
Total		5.54 (2.93)	6.97 (2.98)
Delay to ADHD diagnosis (M, SD)			
Males	3.39 (2.48)		4.01 (2.24)
Females	3.74 (2.73)		4.83 (3.13)
Total	3.49 (2.55)		4.22 (2.51)
Delay to autism diagnosis (M, SD)			
Males		2.75 (2.11)	4.83 (2.68)
Females		3.99 (2.90)	5.30 (3.42)
Total		3.15 (2.45)	4.27 (2.94)

ADHD: attention-deficit/hyperactivity disorder; SD: standard deviation.

Age and diagnostic delay were reported in mean years (SD). Autism = autism spectrum disorder. ADHD + autism = children with co-occurring ADHD and autism. Age at first concern = age at which caregivers first had developmental concerns about their child.

### Age at diagnosis

Children with ADHD received their ADHD diagnosis at 7.52 years (SD = 2.49), which was significantly older than children with ADHD + autism who received their ADHD diagnosis at 6.92 years (SD = 2.35), *F* (1, 517) = 7.52, *p* = 0.006, η_p_^2^ = 0.01.

**Figure 1.**
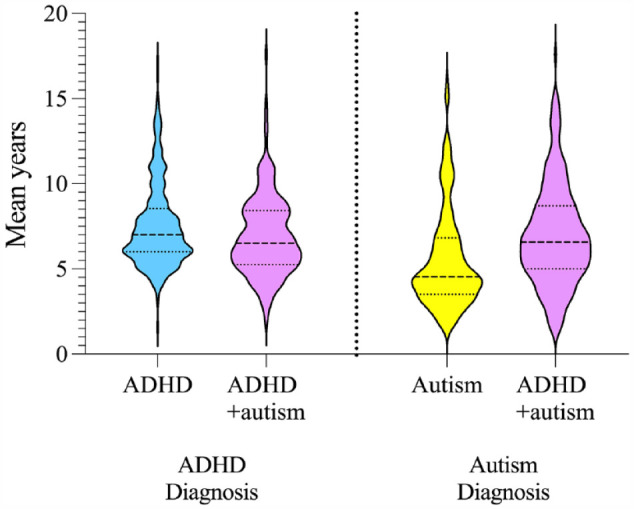
Age at ADHD and autism diagnosis for children with ADHD, autism and ADHD + autism. Age was reported in mean years (SD). ADHD: attention-deficit/hyperactivity disorder; Autism: autism spectrum disorder; ADHD + autism: children with co-occurring ADHD and autism.

Children with autism were significantly younger at autism diagnosis (M = 5.54 years, SD = 2.93) compared to children with ADHD + autism, M = 6.97 years, SD = 2.98, *F* (1, 361) = 20.83, *p* < 0.001, η_p_^2^ = 0.06. See Figure 1 for violin plots of age at ADHD and age at autism diagnosis. See Appendix S5 Table S5 for ANCOVA statistics.

### Diagnostic delay

Mean diagnostic delay for ADHD and autism diagnoses across diagnostic groups and between males and females are provided in Table 2. ANCOVA statistics for delay to ADHD diagnosis for the whole sample are presented in Supplementary Table S6, and delay to ADHD diagnosis between males and females with ADHD in Supplementary Table S7. Results from the exploratory analysis of delay to ADHD and delay to autism diagnosis in the three ADHD + autism groups are presented in Supplementary Appendix S8. Descriptive statistics for age at first concerns, age at ADHD and autism diagnosis, and delay to ADHD and autism diagnosis in the ADHD + autism groups are presented in Supplementary Table S8, and ANCOVA statistics in Supplementary Table S9.

#### Delay to ADHD diagnosis as a function of diagnostic group

**Figure 2.**
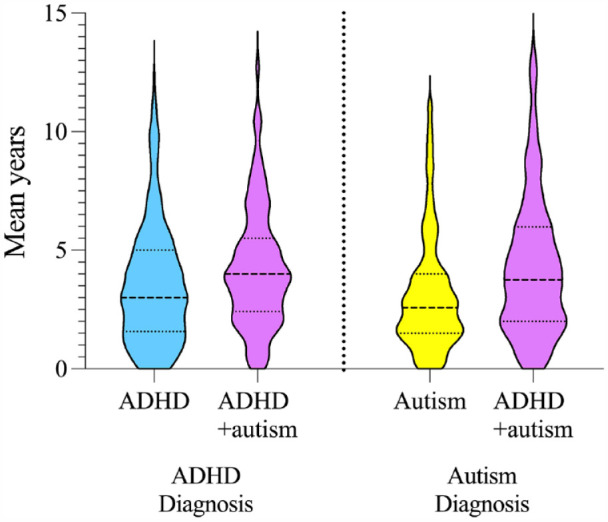
Delay to ADHD and autism diagnosis in years for children with ADHD, autism and ADHD + autism. Age was reported in mean years (SD). ADHD: attention-deficit/hyperactivity disorder; Autism: autism spectrum disorder; ADHD + autism: children with co-occurring ADHD and autism.

After accounting for the effects of child sex, SES and number of co-occurring conditions, the delay between initial general developmental concerns and receiving an ADHD diagnosis was significantly longer for children with ADHD + autism at 4.22 years (SD = 2.51) compared to children with ADHD at 3.49 years, SD = 2.55, *F* (1, 513) = 9.28, *p* = 0.002, η_p_^2^ = 0.02. See Figure 2 for violin plots.

#### Delay to autism diagnosis as a function of diagnostic group

Delay to autism diagnosis was significantly longer for children with ADHD + autism (M = 4.27 years, SD = 2.94) compared to autism only, M = 3.15 years, SD = 2.45, *Welch’s F* (3, 330.87) = 16.09, *p* < 0.001, η^2^ = 0.04. See Figure 2 for violin plots of delay to autism diagnosis.

#### Delay to ADHD diagnosis: males compared to females

**Figure 3.**
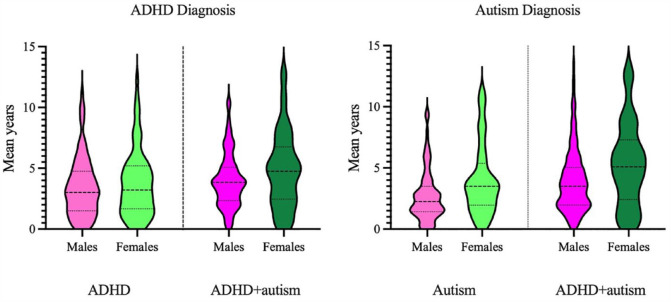
Delay to ADHD and autism diagnosis for males and females with ADHD, autism and ADHD + autism. ADHD: attention-deficit/hyperactivity disorder; Autism: autism spectrum disorder; ADHD + autism: children with co-occurring ADHD and autism.

After accounting for the effects of SES and number of co-occurring conditions, there was no significant difference between males and females with ADHD in delay to ADHD diagnosis, *F* (1, 304) = 1.08, *p* = 0.300, η_p_^2^ = 0.004. Similarly, males and females with ADHD + autism did not significantly differ in delay to ADHD diagnosis, *Welch’s F* (1, 70.65) = 3.10, *p* = 0.083, η^2^ = 0.02. See Figure 3 for violin plots.

#### Delay to autism diagnosis: males compared to females

Females with autism waited 3.99 years (SD = 2.90), which was significantly longer than males (M = 2.75 years, SD = 2.11), *Welch’s F* (1, 72.71) = 7.31, *p* = 0.009, η^2^ = 0.06. Females with ADHD + autism experienced significantly longer delays at 5.30 years (SD = 3.42) to receive an autism diagnosis, which was over a year longer than males (M = 4.83 years, *SD* = 2.68), *Welch’s F* (1, 74,61) = 7.17, *p* = 0.009, η^2^ = 0.04. See Figure 3 for violin plots.

## Discussion

This study examined age at ADHD and autism diagnosis, and diagnostic delay for ADHD and autism diagnoses, in males and females with ADHD, autism and ADHD + autism. Previous work has reported on diagnostic delay in a neurodevelopmental sample (Boulton et al., 2023), or compared children with autism to those with ADHD + autism on age at diagnosis and diagnostic delay for autism diagnosis (Stevens et al., 2016; Wei et al., 2021). However, these studies did not provide estimates for diagnostic delay in males and females across ADHD, autism or ADHD + autism groups specifically.

### Age at diagnosis

The current findings provided baseline ages for when ADHD and autism diagnoses might be expected for children with ADHD + autism. The average age at ADHD and autism diagnosis falls inside previously reported ranges (e.g. Caci et al., 2020; Martinez et al., 2018; Sainsbury et al., 2023). Although the current mean age at autism diagnosis for the autism-only group (5.54 years) was older than previous Australian mean estimates (4 years), it is consistent with previously reported modal values (5.9 years old; Bent et al., 2015). The age at ADHD diagnosis in the current study (7.52 years) is lower than previously reported Australian estimates (Efron et al., 2013), suggesting further confirmation is required. The current findings and other international work align with the ADHD + autism group typically 1.5 years older at autism diagnosis compared to children with autism alone (Kentrou et al., 2019). Similar to Sainsbury et al.’s (2023) recent systematic review, children with ADHD + autism are younger at ADHD diagnosis compared to children with ADHD, and older at autism diagnosis compared to autistic children. A younger age ADHD diagnosis in the ADHD + autism group compared to ADHD only may be linked to elevated ADHD symptom severity in children with ADHD + autism (Elwin et al., 2020).

### Diagnostic delay

As previously reported (Stevens et al., 2016), children with ADHD + autism exhibited longer delays to autism diagnosis compared to autistic children. The current findings demonstrate that children with ADHD + autism also experience longer delays to ADHD diagnosis compared to ADHD-only children. The current delay between first concerns and an ADHD or autism diagnosis for children with ADHD or autism only is consistent with Boulton et al.’s (2023) work, who found a delay of 3.5 years from first concerns to diagnostic assessment. The majority of children in the current study received their ADHD or autism diagnosis privately, compared to Boulton et al.’s (2023) work which focused on a public service. The similarity in delay estimates between the current study and Boulton et al. (2023) suggests that opting for a private diagnostic service may not improve wait times. Although the current results for children with ADHD or autism only align with Boulton et al.’s (2023), our diagnostic delay estimates for children with ADHD + autism are notably longer. This highlights the importance of considering the co-occurring group separately when examining diagnostic delay.

Children with ADHD + autism in the current sample were younger at ADHD diagnosis compared to the ADHD group but experienced significantly longer delays in receiving their ADHD diagnosis. The earlier emergence of developmental concerns in the ADHD + autism group, leading to longer diagnostic delays, may be attributed to autism rather than ADHD symptomatology. However, as the current study did not specifically capture the nature of initial developmental concerns, this warrants future investigation. Previous work tells us that receiving an ADHD diagnosis prior to an autism diagnosis is associated with older age at autism diagnosis (Kentrou et al., 2019; Wei et al., 2021). The current study shows that children with ADHD + autism are not only older but also experience longer delays between initial developmental concerns and receiving a diagnosis of ADHD or autism.

Contrary to expectations, females and males with ADHD and ADHD + autism did not significantly differ in delay to ADHD diagnosis. It is possible that the current study only captured a subgroup of females with ADHD and ADHD + autism who are diagnosed in childhood, missing females who are diagnosed as adults that may have milder symptoms, or predominantly inattentive profiles. Conversely, autistic females and females with ADHD + autism experienced significantly longer delays to autism diagnosis compared to males. Delay to autism diagnosis for autistic females was four years, compared to 2.75 years for males, and similar to trends reported by Siklos and Kerns (2007). However, the diagnostic delay in Siklos and Kerns’ (2007) study was the time from first contact with a clinician to receiving a diagnosis. As the current study included the period of initial developmental concerns, the similarity in estimates may suggest diagnostic delay for females has improved somewhat. Current results for diagnostic delay in females with ADHD + autism were 5.30 years, which is significantly longer than males, and the first-time diagnostic delay for autism has been reported for females with ADHD + autism. As males and females in the ADHD group did not differ on delay to ADHD diagnosis, the longer delay for autistic females and females with ADHD + autism could be, in part, driven by female autistic symptomatology.

There is a delicate balance between timeliness and accuracy when providing diagnosis and intervention. Although no current guideline exists for how long ADHD or autism diagnosis should take, these findings and previous work (Boulton et al., 2023) emphasise the need for improved education and training on the diverse spectrum of ADHD and autism symptoms. Caregivers require education as they may be the first to identify developmental concerns, and specialist training for GP’s, psychologists, and paediatricians has the potential to reduce diagnostic delays. Clinicians should remain cognisant of the high rates of co-occurrence between ADHD and autism, and as ADHD symptoms may in some cases mask autism symptoms, monitoring children diagnosed with one condition for symptoms of the other is recommended (May et al., 2023). This is particularly relevant for females with autism and ADHD + autism, who experienced the longest delays to diagnosis. In future, alternative approaches to care such as the clinical staging approach, where interventions are provided based on functional needs without the need for a diagnosis, could reduce current pressures to provide a diagnosis while still delivering appropriate interventions (Sawrikar et al., 2022).

### Limitations

It is important to note some limitations. First, the study relied on caregiver reports for child diagnosis rather than formal diagnostic interviews. However, caregiver-reported ADHD and autism symptom ratings (see Appendix S1) provided support for caregiver-reported diagnostic groups. Second, although families were required to have a child who received their diagnosis(es) in the last six years to minimise retrospective bias, the retrospective nature of the study could still be subject to potential bias as some caregivers may have reported the age at first developmental concerns for their child as earlier upon reflection. Furthermore, caregivers of older children may have more difficulty recalling when their child’s first developmental concerns arose. The question of diagnostic delay in the presence of co-occurring conditions is complex as different concerns may arise at different stages in a child’s development. The current study utilised general developmental concerns, rather than ADHD or autism-specific concerns, which did not allow for diagnostic delay to be calculated from different time points depending on the type of developmental concerns. Future research building on the current findings would benefit from the use of a prospective design, allowing for the identification of multiple time points for disorder-specific developmental concerns, in turn providing the next steps in understanding diagnostic delay in ADHD, autism, and ADHD + autism. Nevertheless, the results from this large survey sample are largely consistent with previous reports, and additionally identified barriers to diagnosis for females and those with ADHD + autism. Third, this study did not critically examine the specific impact of other co-occurring conditions on diagnostic delay, such as intellectual disability (ID) or learning disorders. The presence of ID in children with autism has been shown to reduce children’s age at diagnosis and diagnostic delay (Stevens et al., 2016). Finally, although the current study has provided valuable insights into diagnostic delay for children and adolescents with ADHD, autism, and ADHD + autism up to 16 years old, this does not capture individuals who have been diagnosed as adults, rates of misdiagnosis or changes in diagnosis. Longitudinal research from childhood to adulthood is needed to capture these aspects of diagnosis across the life span (e.g. Sciberras et al., 2013).

## Conclusion

In some services, a diagnosis is required before children can gain access to funding, support, medication, or early intervention. Not offering services while families wait for a diagnosis can result in delays to intervention and support. Screening for ADHD and autism symptoms in all children presenting with concerns for either disorder will be critical in helping reduce delays to diagnosis and support. The feasibility of a staged approach to clinical care with a focus on current adaptive needs rather than an immediate need for a diagnosis could also reduce pressures associated with receiving a timely diagnosis and subsequent access to intervention (Sawrikar et al., 2022).

Currently, no diagnostic guidelines for identifying children with co-occurring ADHD and autism exist. Instead, guidelines for ADHD point clinicians to the autism guidelines, and vice versa (Australian ADHD Professionals Association, 2022; Whitehouse, et al., 2018; National Institute for Health and Care Excellence (NICE), 2011, 2018), which is problematic. Modifications to future clinical training programmes that highlight the prevalence of co-occurring neurodevelopmental diagnoses, in conjunction with diagnostic and treatment guidelines specifically for the co-occurring ADHD + autism phenotype, especially in females, would likely assist in accelerating the diagnostic process.

## Supplemental Material

sj-docx-1-anp-10.1177_00048674231206997 – Supplemental material for Age at diagnosis and diagnostic delay across attention-deficit hyperactivity and autism spectrums Click here for additional data file. Supplemental material, sj-docx-1-anp-10.1177_00048674231206997 for Age at diagnosis and diagnostic delay across attention-deficit hyperactivity and autism spectrums by Rachael Knott, Olivia J Mellahn, Jeggan Tiego, Kathryn Kallady, Louise E Brown, David Coghill, Katrina Williams, Mark A Bellgrove and Beth P Johnson in Australian & New Zealand Journal of Psychiatry

sj-docx-2-anp-10.1177_00048674231206997 – Supplemental material for Age at diagnosis and diagnostic delay across attention-deficit hyperactivity and autism spectrums Click here for additional data file. Supplemental material, sj-docx-2-anp-10.1177_00048674231206997 for Age at diagnosis and diagnostic delay across attention-deficit hyperactivity and autism spectrums by Rachael Knott, Olivia J Mellahn, Jeggan Tiego, Kathryn Kallady, Louise E Brown, David Coghill, Katrina Williams, Mark A Bellgrove and Beth P Johnson in Australian & New Zealand Journal of Psychiatry
