# Supplementary material for: Age at diagnosis and diagnostic delay across attention-deficit hyperactivity and autism spectrums
Source: Aust N Z J Psychiatry. 2023 Oct 27;58(2):142–51. doi: 10.1177/00048674231206997 (PMC10838471; doi:10.1177/00048674231206997)
Supplement: sj-docx-1-anp-10.1177_00048674231206997 – Supplemental material for Age at diagnosis and diagnostic delay across attention-deficit hyperactivity and autism spectrums [file sj-docx-1-anp-10.1177_00048674231206997.docx]

**Supplemental material title:**

Supplemental material for the ‘Age at diagnosis and diagnostic delay across attention deficit hyperactivity and autism spectrums’ manuscript

**Supplemental material description:**

A subset of the main sample completed ADHD and autism symptoms rating scales, and scores are reported for the ADHD, autism, and ADHD+autism groups. Significance testing between the ADHD, autism, and ADHD+autism is also reported in the supplemental material. Main effect ANCOVA statistics are included for all ANCOVA analyses described in the main manuscript.

Rachael Knott grants the Royal Australian and New Zealand College of Psychiatrists a non-exclusive worldwide licence to reproduce and publish the aforementioned material as supplemental material to Australian and New Zealand Journal of Psychiatry in the English language in all print and electronic formats of Australian and New Zealand Journal of Psychiatry for the life of Australian and New Zealand Journal of Psychiatry, including any future Royal Australian and New Zealand College of Psychiatrists print and electronic media, formats and products which may include Australian and New Zealand Journal of Psychiatry in its entirety. Full copyright acknowledgement will be made to the rights holder for this use.
