# Supplementary material for: Age at diagnosis and diagnostic delay across attention-deficit hyperactivity and autism spectrums
Source: Aust N Z J Psychiatry. 2023 Oct 27;58(2):142–51. doi: 10.1177/00048674231206997 (PMC10838471; doi:10.1177/00048674231206997)
Supplement: sj-docx-2-anp-10.1177_00048674231206997 – Supplemental material for Age at diagnosis and diagnostic delay across attention-deficit hyperactivity and autism spectrums [file sj-docx-2-anp-10.1177_00048674231206997.docx]

**Supplemental material for ‘Age at diagnosis and diagnostic delay across attention deficit hyperactivity and autism spectrums’**

# Appendix S1.

## Table S1

*Frequencies for recruitment by Australian state and the diagnostic setting in which children received their diagnosis*

| Frequencies | ADHD  *N* = 310 | Autism  *N* = 154 | ADHD+ autism  *N* = 213 | Total sample  *N* = 677 |
| --- | --- | --- | --- | --- |
| Australian State |  |  |  |  |
| Victoria | 107 | 63 | 88 | 258 |
| NSW | 105 | 32 | 55 | 192 |
| Queensland | 50 | 28 | 45 | 123 |
| Western Australia | 21 | 11 | 2 | 34 |
| South Australia | 6 | 8 | 11 | 25 |
| Tasmania | 9 | 3 | 3 | 15 |
| ACT | 9 | 8 | 9 | 26 |
| Northern Territory | 3 | 1 | 0 | 4 |
| **Diagnostic setting: ADHD** |  |  |  |  |
| Public setting (total) | 50 |  | 35 | 85 |
| Public health | 30 |  | 26 | 56 |
| Community health | 20 |  | 9 | 29 |
| Private setting (total) | 259 |  | 178 |  |
| Private paediatrician | 173 |  | 115 | 288 |
| Private psychologist | 47 |  | 24 | 71 |
| Private psychiatrist | 4 |  | 8 | 12 |
| Private MDT | 35 |  | 31 | 66 |
| Private allied health | 0 |  | 0 | 0 |
| Not sure | 0 |  | 0 | 0 |
| **Diagnostic setting: Autism** |  |  |  |  |
| Public setting (total) |  | 25 | 49 | 74 |
| Public health |  | 18 | 34 | 52 |
| Community health |  | 7 | 15 | 22 |
| Private setting (total) |  | 128 | 162 | 290 |
| Private paediatrician |  | 43 | 41 | 84 |
| Private psychologist |  | 38 | 41 | 79 |
| Private psychiatrist |  | 1 | 7 | 8 |
| Private MDT |  | 41 | 72 | 113 |
| Private allied health |  | 2 | 0 | 2 |
| Not sure |  | 3 | 1 | 4 |

*Note.* ADHD = attention deficit/hyperactivity disorder. Autism = autism spectrum disorder. ADHD+autism = co-occurring attention deficit/hyperactivity disorder and autism spectrum disorder. NSW = New South Wales. ACT = Australian Capital Territory. MDT = multi-disciplinary team.

# Appendix S2.

A subset of the sample (*N* = 553) also completed ADHD and autism symptom rating scales, the Conners’ Parent Rating Scale – Revised Long Form (CPRS) and the Social Responsiveness Scale – Second Edition (SRS-2).

**Parent rated ASD and ADHD symptom scales at the time of survey**

**Social Responsiveness Scale – Second Edition (SRS-2).** The Social Responsiveness Scale – Second Edition (SRS-2) is a 65-item parent-report measure for children aged four to 18 years of age (Constantino, 2011). Items are rated on a 4-point Likert scale and index social and behavioural difficulties associated with autism. Raw scores are converted to age and gender matched *T*-scores for the DSM-V subscales, which are the social communication index (SCI) and restricted and repetitive behaviours (RRB), and the total score. Higher *T*-scores indicate a greater level of social or behavioural difficulty.

**Conners’ Parent Rating Scale – Revised Long Form (CPRS-RL).** The Conners’ Parent Rating Scale – Revised Long Form is an 80-item parent-reported measure for behaviours associated with ADHD, including inattention, hyperactivity, and impulsivity (Conners, Sitarenios, Parker, & Epstein, 1998). Items are rated on a 4-point Likert scale, with raw scores converted to age and gender matched *T*-scores. The DSM-IV subscales for inattentive and hyperactive impulsive were used, as well as the CPRS total score.

**Significance Testing Results**

**SRS-2 and CPRS *T*-scores for the ASD, ADHD, and ADHD+autism groups**

A subset of the sample (*N* = 553, ASD = 129, ADHD = 260, ADHD+autism = 164) completed ASD and ADHD symptom rating scales. See Table A1 for means and standard deviations for SRS-2 and CPRS scores for ASD, ADHD, and ADHD+autism groups.

With respect to ASD symptoms, three Kruskal-Wallis ANOVAs indicated that there was a significant different between ASD, ADHD, and ADHD+autism groups on SRS-2 RRB subscale scores, *H* = 116.71, *df* = 2, *N* = 555, *p* < .001, SRS-2 SCI subscale scores, *H* = 138.76, *df* = 2, *N* = 555, *p* < .001, and SRS-2 Total scores, *H* = 148.86, *df* = 2, *N* = 551, *p* < .001. Nine post-hoc Mann-Whitney *U* tests were used to determine which groups significantly differed on ASD symptom ratings. Significance was assessed with a Bonferroni adjusted alpha for multiple comparisons (*α* = .005). Mann-Whitney *U* tests indicated that the ASD group had significantly higher RRB scores, *U* = 8612.50, *z* = -7.83, *p* < .001, SCI scores, *U* = 726.50, *z* = -9.11, *p* < .001, and SRS-2 total scores, *U* = 7126.00, *z* = -9.25, *p* < .001, compared to the ADHD group. The ASD and ADHD+autism groups did not significantly differ on RRB scores, *U* = 9740.50, *z* = -1.35, *p* = .178, SCI scores, *U* = 10471.00, *z* = -0.33, *p* = .745, and SRS-2 total scores, *U* = 10134.00, *z* = -0.79, *p* = .426. The ADHD had significantly lower RRB scores, *U* = 9579.50, *z* = -9.71, *p* < .001, SCI scores, *U* = 8911.59, *z* = -10.23, *p*< .001, and SRS-2 total scores, *U* = 8334.00, *z* = -10.71, *p* < .001, compared to the ADHD+autism group.

Three Kruskal-Wallis ANOVAs showed the ASD, ADHD, and ADHD+autism groups significantly differed on ADHD symptoms, that is, CPRS inattentive subscale scores, *H* = 148.86, *df* = 2, *N* = 551, *p* < .001, CPRS hyperactive/impulsive subscale scored, *H* = 23.55, *df* = 2, *N* = 552, *p* < .001, and CPRS total scores, *H* = 23.91, *df* = 2, *N* = 551, *p* < .001. Nine post-hoc Mann-Whitney *U* tests were used to determine which groups significantly differed on ADHD symptom ratings. Significance was assessed with a Bonferroni adjusted *p*-value for multiple comparisons (*α* = .005). Mann-Whitney *U* tests indicated that the ADHD group had significantly higher inattentive scores, *U* = 12849.00, *z* = -3.44, *p* = .001, hyperactive/impulsive scores, *U* = 12551.50, *z* = -3.84, *p* < .001, and CPRS total scores, *U* = 12138.00, *z* = -4.14, *p* < .001, compared to the ASD group. The ADHD+autism group had significantly higher inattentive scores, *U* = 7892.50, *z* = -3.52, *p* < .001, hyperactive/impulsive scores, *U* = 7039.00, *z* = -4.82, *p* < .001, and CPRS total scores, *U* = 7070.00, *z* = -4.68, *p* < .001, compared to the ASD only group. The ADHD and ADHD+autism groups did not significantly differ on inattentive scores, *U* = 20996.50, *z* = -0.37, *p* = .713, hyperactive/impulsive scores, *U* = 20120.50, *z* = -1.08, *p* = .279, and CPRS total scores, *U* = 20656.00, *z* = -0.65, *p* = .518.

## Table S2

*SRS-2 and CPRS T-Scores for Children with Caregiver-Reported ADHD, Autism, and ADHD+autism.*

|  | ADHD | Autism | ADHD+autism |
| --- | --- | --- | --- |
|  | *M* (*SD*) | *M* (*SD*) | *M* (*SD*) |
| SRS-2 |  |  |  |
| RRB Subscale | 68.37 (12.41) | 79.03 (10.14) | 80.61 (9.56) |
| SCI Subscale | 65.88(11.47) | 77.55 (9.19) | 78.13 (9.22) |
| Total | 68.41 (12.04) | 80.77 (8.94) | 81.64 (8.43) |
| CPRS |  |  |  |
| Inattentive | 74.15 (10.58) | 70.25 (11.29) | 74.76 (9.83) |
| Hyp/Imp | 75.71 (12.89) | 70.56 (12.40) | 77.47 (11.29) |
| Total | 77.00 (11.09) | 72.04 (10.96) | 77.98 (9.47) |

*Note.* ADHD = Attention Deficit Hyperactivity Disorder. Autism = Autism Spectrum Disorder. ADHD+autism = when ADHD and autism co-occur. SRS-2 = Social Responsiveness Scale – Second Edition. RRB = Restricted and repetitive behaviours. SCI = Social communication index. CPRS = Conners’ Parent Rating Scale – Revised Long Form. Hyp/Imp = Hyperactive/impulsive subscale. Inattentive = Inattentive subscale.

# Appendix S3.

Caregivers completed a checklist of autism and/or ADHD symptoms, depending on their child’s diagnosis, to indicate which symptoms were present when they first had developmental concerns about their child. See below for ADHD and autism symptom checklists.

## ADHD Symptoms:

1. Fail to pay close attention to details or make careless mistakes in schoolwork
2. Have trouble staying focused in tasks or play
3. Appear not to listen, even when spoken to directly
4. Have difficulty following through on instructions and fail to finish schoolwork or chores
5. Have trouble organizing tasks and activities
6. Avoid or dislike tasks that require focused mental effort, such as homework
7. Lose items needed for tasks or activities, for example, toys, school assignments, pencils
8. Be easily distracted
9. Forget to do some daily activities, such as forgetting to do chores
10. Fidget with or tap his or her hands or feet, or squirm in the seat
11. Have difficulty staying seated in the classroom or in other situations
12. Be on the go, in constant motion
13. Run around or climb in situations when its not appropriate
14. Have trouble playing or doing an activity quietly
15. Talk too much
16. Blurt out answers, interrupting the questioner
17. Have difficulty waiting for his or her turn
18. Interrupt or intrude on others conversations, games or activities

## Autism Symptoms:

1. Performs repetitive movements, such as rocking, spinning or hand flapping
2. Performs activities that could cause self-harm, such as biting or head-banging
3. Develops specific routines or rituals and becomes disturbed at the slightest change
4. Has problems with coordination or has odd movement patterns, such as clumsiness or walking on toes, and has odd, stiff or exaggerated body language
5. Is fascinated by details of an object, such as the spinning wheels of a toy car, but doesn’t understand the overall purpose or function of the object
6. Is unusually sensitive to light, sound or touch, yet may be indifferent to pain or temperature
7. Doesn’t engage in imitative or make-believe play
8. Fixates on an object or activity with abnormal intensity or focus
9. Has specific food preferences, such as eating only a few foods, or refusing foods with a certain texture
10. Fails to respond to his or her name or appears not to hear you at times
11. Resists cuddling and holding, and seems to prefer playing alone, retreating into his or her own world)
12. Has poor eye contact and lacks facial expression
13. Doesn’t speak or has delayed speech, or loses previous ability to say words or sentences
14. Cant start a conversation or keep one going, or only starts one to make requests or label items
15. Speaks with an abnormal tone or rhythm and may use a singsong voice or robot-like speech
16. Doesn’t appear to understand simple questions or directions
17. Doesn’t express emotions or feelings and appears unaware of others feelings
18. Doesn’t point at or bring objects to share interest
19. Inappropriately approaches a social interaction by being passive, aggressive or disruptive
20. Has difficulty recognizing nonverbal cues, such as interpreting other peoples facial expressions, body postures or tone of voice

**Results**

**Symptoms Present at First Concerns**

Caregivers’ responses to the ADHD and autism behaviour checklists were informally examined. No notable differences were observed between caregivers of children with ADHD and ADHD+autism in their endorsement of ADHD symptoms. “Having trouble staying focused in tasks or play”, “appearing not to listen”, and “having difficulty following instructions” were among the most frequently rated symptoms in both groups. Responses to the autism behaviour checklist were similar for caregivers of children with autism and ADHD+autism, although a slight trend towards ADHD+autism caregivers endorsing more symptoms that would fall in the restrictive and repetitive behaviours (RRBs), interests, and activities category (e.g., “3. Develops specific routines or rituals and becomes disturbed at the slightest change” and “9. Has specific food preferences”). Qualitatively, these results suggest that both ADHD and autism symptoms were developmental concerns for children with ADHD+autism. See Figure S1 and S2 for the percent of caregivers who endorsed each ADHD and autism symptom. Symptoms were not mutually exclusive.

## Figure S1

*ADHD Symptom Checklist Completed by Caregivers for Behaviours Present when General Developmental Concerns Arose*

*Note.* The items on the checklist are not mutually exclusive. Percentages are calculated within each diagnostic group, for example, 62% of caregivers of children with ADHD endorsed ‘1. Fail to pay close attention’.

## Figure S2

*Autism Symptom Checklist Completed by Caregivers for Behaviours Present when General Developmental Concerns Arose*

*Note.* The items on the checklist are not mutually exclusive. Percentages are calculated within each diagnostic group, for example, 43% of caregivers of children with autism only endorsed ‘1. Repetitive movements’.

# Appendix S4.

Correlations of the covariates (SES, number of additional co-occurring conditions) with age at diagnosis for ADHD and autism, diagnostic delay for ADHD, and diagnostic delay for autism.

## Table S4

*Pearson correlations of the covariates SES and number of additional co-occurring conditions with age at diagnosis for ADHD and autism, and diagnostic delay for ADHD and autism*

| Pearson correlations | 1. | 2. | 3. | 4. | 5. | 6. |
| --- | --- | --- | --- | --- | --- | --- |
| 1. SES | 1 | .04 | .07 | .04 | .05 | -.01 |
| 1. No. co-occurring conditions |  | 1 | .15** | .02 | .16** | .03* |
| 1. Age at ADHD diagnosis |  |  | 1 | .61** | .44** | .61** |
| 1. Age at autism diagnosis |  |  |  | 1 | .79** | .34** |
| 1. Delay to ADHD diagnosis |  |  |  |  | 1 | .61** |
| 1. Delay to autism diagnosis |  |  |  |  |  | 1 |

*Note.* SES = socio-economic status. No. co-occurring conditions = number of additional co-occurring conditions. ADHD = attention deficit/hyperactivity disorder. Autism = autism spectrum disorder.

* *p*-value < .05. ** *p*-value < .001.

# Appendix S5.

One-way between groups analysis of covariance (ANCOVA) statistics for age at ADHD and age at autism diagnosis. Sex, SES, and number of additional co-occurring conditions were entered as covariates in both analyses.

## Table S5

*One-Way ANCOVA Statistics for Age at ADHD and Autism Diagnosis Across Diagnostic Groups.*

| Results | *df* | *SS* | *MS* | *F* |
| --- | --- | --- | --- | --- |
| Age at ADHD Diagnosis |  |  |  |  |
| Intercept | 1 | 284804.72 | 284804.72 | 338.53** |
| No. co-occurring | 1 | 302.95 | 302.95 | 0.36 |
| Sex | 1 | 6988.34 | 6988.34 | 8.31* |
| SES | 1 | 25.54 | 25.54 | 0.30 |
| Diagnostic group | 1 | 6327.83 | 6327.83 | 7.52* |
| Error | 517 | 434954.09 | 841.30 |  |
| Total | 522 | 4426211.00 |  |  |
| Age at Autism Diagnosis |  |  |  |  |
| Intercept | 1 | 127227.56 | 127227.56 | 106.99** |
| No. co-occurring | 1 | 1946.63 | 1946.63 | 1.64 |
| Sex | 1 | 22728.21 | 22728.21 | 19.11** |
| SES | 1 | 1305.15 | 1305.15 | 1.09 |
| Diagnostic group | 1 | 24764.19 | 24764.19 | 20.83** |
| Error | 670 | 429269.79 | 1189.11 |  |
| Total | 676 | 2622145.00 |  |  |

*Note.* Biological sex, SES, and number of co-occurring diagnoses entered as covariates. *df* = degrees of freedom. *SS* = Type III Sum of Squares. *MS* = Mean Square. *F* = *F*-statistic. ANCOVA = Analysis of covariance. No. co-occurring = number of co-occurring diagnoses. Sex = child’s biological sex. SES = socio economic status.

* *p* < .05. ** *p* < 0.001.

# Appendix S6

One-way between groups analysis of covariance (ANCOVA) statistics for delay to ADHD diagnosis between ADHD and ADHD+autism groups.

## Table S6

*One-way Between Groups ANCOVA Statistics for Delay to ADHD Diagnosis.*

| ANCOVAs | *df* | *SS* | *MS* | *F* |
| --- | --- | --- | --- | --- |
| Delay to ADHD Diagnosis |  |  |  |  |
| Intercept | 1 | 78176.54 | 78176.54 | 4.53* |
| No. co-occurring | 1 | 2617.33 | 2617.33 | 85.32** |
| Sex | 1 | 3662.19 | 3662.19 | 2.86 |
| SES | 1 | 68.78 | 68.78 | 3.99* |
| Diagnostic group | 1 | 8501.97 | 8501.97 | 9.28* |
| Error | 513 | 470031.35 | 916.24 |  |
| Total | 518 | 1558008.00 |  |  |

*Note.* ANCOVA = Analysis of covariance. Child biological sex, SES and number of co-occurring diagnoses entered as covariates. *df* = degrees of freedom. *SS* = Type III Sum of Squares. *MS* = Mean Square. *F* = *F*-statistic. No. co-occurring = number of co-occurring diagnoses. Sex = child’s biological sex. SES = socio economic status.

* *p* < .05. ** *p* < 0.001.

# Appendix S7

One-way between groups analysis of covariance (ANCOVA) statistics for delay to ADHD diagnosis between males and females with ADHD only.

## Table S7

*One-way ANCOVA Statistics for Delay to ADHD Diagnosis between Males and Females with ADHD.*

|  | *df* | *SS* | *MS* | *F* |
| --- | --- | --- | --- | --- |
| ADHD only |  |  |  |  |
| Intercept | 1 | 40228.76 | 40228.76 | 43.33** |
| No. co-occurring | 1 | 3114.37 | 3114.37 | 3.36 |
| SES | 1 | 331.73 | 331.73 | 0.36 |
| Child Sex | 1 | 1001.12 | 1001.12 | 1.08 |
| Error | 304 | 282236.47 | 928.41 |  |
| Total | 308 | 826519.00 |  |  |

*Note.* ANCOVA = Analysis of covariance. Number of co-occurring conditions and SES entered as covariates. *df* = degrees of freedom. *SS* = Type III Sum of Squares. *MS* = Mean Square. *F* = *F*-statistic. No. co-occurring = number of co-occurring diagnoses. Sex = child’s biological sex. SES = socio economic status.

** *p* < 0.001.

# Appendix S8

## Diagnostic delay for ADHD+autism children only

Two ANCOVAs were performed for to examine differences in delay to ADHD and autism diagnoses between the three ADHD+autism subgroups, with SES, child sex, and number of co-occurring conditions as covariates. However, as homogeneity of variance could not be assumed for delay to autism diagnosis (*F* (2, 207) = 4.90, *p* = .008) a one-way between groups ANOVA was performed using Welch’s *F* statistic ([Field, 2013](#_ENREF_15)). The age at first general developmental concerns, age at ADHD and autism diagnoses, and diagnostic delay are plotted in Figure S3 for the three ADHD+autism subgroups.

Of the children with ADHD+autism 33.33% (*N* = 71) received their ADHD diagnosis first, 35.21% (*N* = 75) received their autism diagnosis first, and 31.46% (*N* = 67) received their ADHD and autism diagnoses at the same time. See Table S8 for means years and standard deviations of age at first concern, age at ADHD and autism diagnosis, and diagnostic delay estimates, and Table S9. Overall, these findings suggest that diagnostic overshadowing may be particularly salient in a sub-group of children with ADHD+autism who receive their ADHD diagnosis first.

**Delay to ADHD diagnosis: ADHD+autism children only**

There was a significant main effect of ADHD+autism subgroup (*F* (2, 204) = 6.44, *p* = .002, η_p_^2^ = .06). Post-hoc comparisons indicated children with ADHD+autism who received their autism diagnosis first experienced significantly longer delays to receiving an ADHD diagnosis (*M* = 4.92 years, *SD* = 2.36, 95%) compared to children with ADHD+autism who received their ADHD diagnosis first (*M* = 3.65 years, *SD* = 2.25), *p* = .002, 95% CI [0.46, 2.45]. Children who received their ADHD and autism diagnoses at the same time did not differ from children who received their ADHD, *p* = .674, 95% CI [-0.51, 1.54], or autism diagnosis first, *p* = .079, 95% CI [-1.95, 0.07].

**Delay to autism diagnosis: ADHD+autism children only**

The three ADHD+autism subgroups significantly differed on delay to autism diagnosis, *Welch’s* *F* (2, 129.88) = 32.49, *p* < .001, *η^2^* = .23. Children with ADHD+autism who received their ADHD diagnosis first experienced significantly longer delays (*M* = 6.11 years, *SD* = 2.95) to receive an autism diagnosis compared to children with ADHD+autism who received their autism diagnosis first (*M* = 2.74 years, *SD* = 1.99), *p* < .001, 95% CI [2.33, 4.40], and children with ADHD+autism who received their ADHD and autism diagnoses at the same time (*M* = 4.03 years, *SD* = 2.78), *p* < .001, 95% CI [1.01, 3.15].

## Figure S3

*Age at First Concerns, Age at Autism Diagnosis, and Age at ADHD Diagnosis for Children with ADHD+autism*


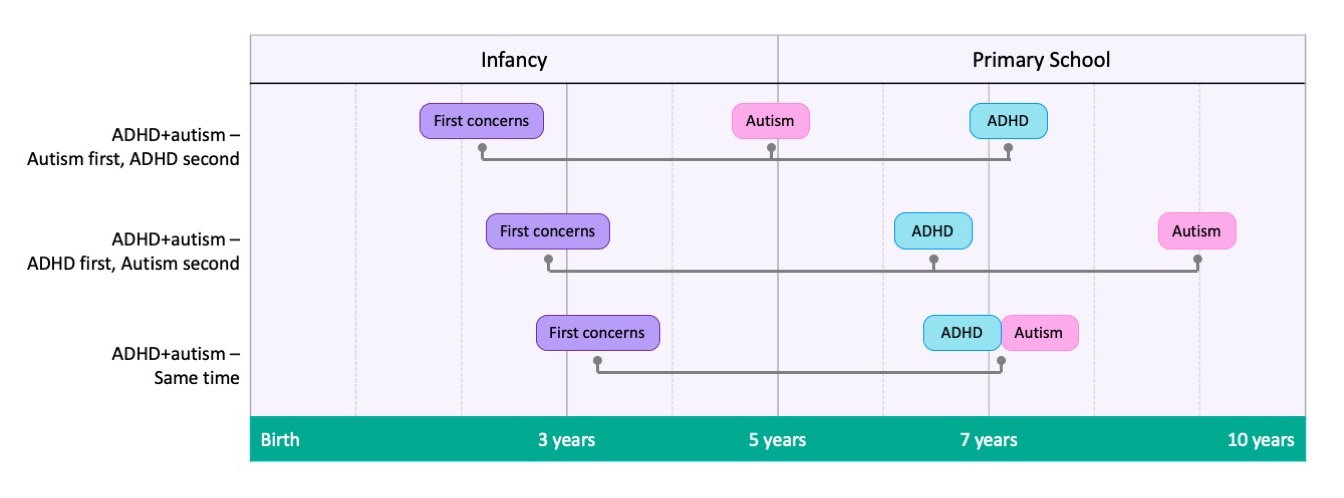


*Note.* ADHD = attention deficit/hyperactivity disorder. Autism = autism spectrum disorder. ADHD+autism = co-occurring attention deficit/hyperactivity disorder and autism spectrum disorder. ADHD+autism – ADHD first = children with ADHD+autism who received their ADHD diagnosis before autism. ADHD+autism – autism first = children with ADHD+autism who received their autism diagnosis before ADHD. ADHD+autism – same time = children with ADHD+autism who received their ADHD and autism diagnoses at the same time. Age at first concern = age at which caregivers first had general developmental concerns about their child.

## Table S8

*Mean Age in Years at First Developmental Concern, Age at ADHD and Autism Diagnosis, and Delay to ADHD and Autism Diagnosis for the ADHD+Autism Groups*

|  | ADHD+autism  – ADHD first  *N* = 71 | ADHD+autism  – autism first  *N* = 75 | ADHD+autism  – same time  *N* = 67 |
| --- | --- | --- | --- |
| Age at first concern (*M, SD*) |  |  |  |
| Males | 2.82 (1.75) | 2.02 (1.49) | 3.40 (1.82) |
| Females | 2.81 (1.43) | 3.07 (2.53) | 2.66 (2.07) |
| Total | 2.82 (1.64) | 2.20 (1.74) | 3.21 (1.89) |
| Age at ADHD Diagnosis (*M, SD*) |  |  |  |
| Males | 6.22 (2.04) | 6.79 (1.98) | 7.03 (2.66) |
| Females | 6.97 (1.89) | 8.71 (2.39) | 7.55 (3.21) |
| Total | 6.47 (2.01) | 7.13 (2.16) | 7.16 (2.79) |
| Age at Autism Diagnosis (*M, SD*) |  |  |  |
| Males | 8.70 (2.66) | 4.67 (1.72) | 7.03 (2.66) |
| Females | 9.35 (2.56) | 6.22 (2.84) | 7.55 (3.21) |
| Total | 8.92 (2.63) | 4.94 (2.02) | 7.16 (2.79) |
| Delay to ADHD Diagnosis (*M, SD*) |  |  |  |
| Males | 3.39 (2.17) | 4.78 (2.2) | 3.64 (2.11) |
| Females | 4.15 (2.36) | 5.63 (3.00) | 5.19 (4.11) |
| Total | 3.65 (2.25) | 4.92 (2.36) | 4.03 (2.78) |
| Delay to Autism Diagnosis (*M, SD*) |  |  |  |
| Males | 5.88 (3.09) | 2.65 (1.78) | 3.64 (2.11) |
| Females | 6.54 (2.66) | 3.15 (2.82) | 5.19 (4.11) |
| Total | 6.11 (2.95) | 2.74 (1.99) | 4.03 (2.78) |

*Note.* Age and diagnostic delay reported in mean years (*SD*). ADHD = attention deficit/hyperactivity disorder. Autism = autism spectrum disorder. ADHD+autism = children with co-occurring ADHD and autism. Age at first concern = age at which caregivers first had developmental concerns about their child.

## Table S9

*One-way ANCOVA Statistics for Delay to ADHD Diagnosis Across the Three ADHD+autism Subgroups.*

|  | *df* | *SS* | *MS* | *F* |
| --- | --- | --- | --- | --- |
| Delay to ADHD Diagnosis |  |  |  |  |
| Intercept | 1 | 39304.38 | 39304.38 | 45.81** |
| No. co-occurring | 1 | 295.19 | 295.19 | 0.34 |
| SES | 1 | 9.10 | 9.10 | 0.01 |
| Child Sex | 1 | 5479.98 | 5479.98 | 6.39 |
| ADHD+autism group | 2 | 11056.55 | 5528.27 | 6.44* |
| Error | 204 | 175047.27 | 858.08 |  |
| Total | 210 | 731489.00 |  |  |

*Note.* ANCOVA = Analysis of covariance. Number of co-occurring conditions and SES entered as covariates. *df* = degrees of freedom. *SS* = Type III Sum of Squares. *MS* = Mean Square. *F* = *F*-statistic. No. co-occurring = number of co-occurring diagnoses. Sex = child’s biological sex. SES = socio economic status. ADHD+autism group = the three ADHD+autism subgroups; 1) ADHD+autism – ADHD first, 2) ADHD+autism – autism first, 3) ADHD+autism – same time.

* *p* < .05. ** *p* < 0.001.

**References**

Conners, C. K., Sitarenios, G., Parker, J. D. A., & Epstein, J. N. (1998). The revised Conners' Parent Rating Scale (CPRS-R): Factor structure, reliability, and criterion validity. *Journal of Abnormal Child Psychology, 26*(4), 257-268.

Constantino, J. N. (2011). *Social Responsiveness Scale, Second Edition (SRS-2)*. Torrence, CA: Western Psychological Services.
